# Forgetting how we ate: personalised nutrition and the strategic uses of history

**DOI:** 10.1007/s40656-024-00613-x

**Published:** 2024-03-07

**Authors:** Christopher Mayes, Maurizio Meloni

**Affiliations:** School of Humanities and Social Sciences, Deakin University, Geelong, Australia

**Keywords:** Personalised nutrition, Postgenomics, History of nutrition, Humoralism, Food ethics

## Abstract

Personalised nutrition (PN) has emerged over the past twenty years as a promising area of research in the postgenomic era and has been popularized as the new big thing out of molecular biology. Advocates of PN claim that previous approaches to nutrition sought general and universal guidance that applied to all people. In contrast, they contend that PN operates with the principle that “one size does not fit all” when it comes to dietary guidance. While the molecular mechanisms studied within PN are new, the notion of a personal dietary regime guided by medical advice has a much longer history that can be traced back to Galen’s “On Food and Diet” or Ibn Sina’s (westernized as Avicenna) “Canon of Medicine”. Yet this history is either wholly ignored or misleadingly appropriated by PN proponents. This (mis)use of history, we argue helps to sustain the hype of the novelty of the proposed field and potential commodification of molecular advice that undermines longer histories of food management in premodern and non-Western cultures. Moreover, it elides how the longer history of nutritional advice always happened in a heavily moralized, gendered, and racialized context deeply entwined with collective technologies of power, not just individual advice. This article aims at offering a wider appreciation of this longer history to nuance the hype and exceptionalism surrounding contemporary claims.

**Supplementary Information:**

The online version contains supplementary material available at 10.1007/s40656-024-00613-x.

## Introduction

Personalised nutrition (PN) has emerged over the past twenty years as a promising area of research in the postgenomic era and has been popularized by scientific and cultural entrepreneurs as the new big thing out of molecular biology. For example, in September 2022, Lykon, a Berlin-based food and health tech company, secured €10 million from venture capitalists to develop AI-driven personalised nutrition products. The developers claim that with “just a few drops of blood or saliva, which can be collected at home, [they can] provide customers with tailored nutrition and action recommendations to help them achieve their health goals” (Paul, 2022). On Lykon’s homepage is the text, “An apple a day is not for everyone” accompanied by images of two women, Emma and Simone. For Emma, the apple purportedly is a “superfood” to help lose weight, while for Simone the apple leads to “blood sugar peak” and will not contribute to weight loss (Lykon, 2022). The idea that the same food item, in this case an apple, can have dramatically different health effects for different individuals is central to PN’s claim of novelty and effectiveness. Our goal in this article is to critically evaluate this claim and investigate how it relates to the much longer history of nutrition and dietary advice.

PN is positioned as a contrast to a universal diet applicable to all people. In particular, proponents of PN seek to differentiate themselves from ‘modernist’ nutrition science that has its roots in mid-19th C Europe (Cannon, 2005). The emergence of nutrition science was made possible due to the nascent fields of chemical physiology and biochemistry that enabled general and universal dietary guidance that purportedly applied to all people (Kamminga & Cunningham, 1995). However, the translation of this knowledge into the daily lives of individuals and communities was not so straightforward and it helped to formalise ancient or humoral dietetics into home economics and then the profession of dieticians (Cannon, 2005; Brady, 2018). There is longstanding tension between nutrition science and dietetics regarding status and authority of dietary advice (Shapin, 2014). We are not going to be able to fully explore this history but it is worth noting some of the points of tension. Firstly, cultural understandings of food and health did not simply cede to the march of nutrition science. Dieticians had a role in assisting communities adapt and modify nutrition advice to meet dietary, cultural, and culinary needs. This did not always align with the perspectives of nutrition scientists. Secondly, dieticians were able to gain some authority due to their association with medical or clinical practice. Dieticians could assist clinical objectives as mediators who had access to peoples’ kitchens, homes, and daily lives (DeVault, 1995). In the late-19th C dietitians emerged to translate and tailor scientific knowledge to the daily lives and dietary needs of specific cohorts, such as pregnant women or children (Brady, 2017, 2018).

PN, however, in its rush to claim novelty ignores this history of tailoring in general, and the role of dietitians in particular. PN advocates emphasise that it operates with the principle that “one size does not fit all” when it comes to dietary guidance. Each individual has a unique genetic, epigenetic and microbial profile with specific nutritional needs. There is no agreed definition of PN but since the early 2000s it is broadly associated with a cluster of genomic and epigenomic knowledges examining the way an individual’s diet influences gene expression and/or their gut-microbiome (Simon et al., 2023). This research is increasingly proposed by public health nutritionists to make targeted nutritional advice and interventions to prevent disease and improve health in an individual (Mathers, 2019; Marks et al., 2020).

While the molecular mechanisms studied within PN are new, the notion of a personal dietary regime guided by medical advice is not. The idea that an individual’s diet is directly associated with their unique humoral balance, at different time of the day, season or life, has a much longer history that can be traced back to Galen’s “On Food and Diet” (Grant, 2002) or Ibn Sina’s (westernized as Avicenna) “Canon of Medicine” (Abu-Asab et al., 2013), the key medical textbook of premodern Europe until the 16th century (Siraisi, 2014). Yet this history is either quarantined or misleadingly appropriated by PN proponents. Some proponents of PN pair claims of PN’s novelty with the Hippocratic humoral tradition, including apocryphal idioms such as “let thy food be thy medicine” (Cardenas, 2013). This (mis)use of history, we argue, is not just the result of academic silos and “strategic ignorance” among disciplines (McGoey, 2012). Rather, it is helpful to sustain the hype of the novelty of the proposed field and its potential commodification of molecular advice that undermines longer histories of food management in premodern and traditional cultures. This is a case of non-transferred knowledge in the sense of Proctor and Schiebinger’s *Agnotology* (2008), a cultivated ignorance that underpins a certain political economy of knowledge based on hype, radical novelty of the present, and elision of the heavily moralized, classed, gendered and racialized context of traditional nutritional advice (Earle, 2010, 2012, 2019).

Combining sociological methods and historical work, this article aims at offering a wider appreciation of this longer history to nuance the hype and exceptionalism surrounding contemporary claims and reveal the complex pathways through which biological determinism has historically exceeded reference to genetic factors. While the aura of novelty and appeal to environmental, hence modifiable factors, warrants more political credibility to PN, we show how its postgenomic imagination relies on simplistic causal accounts about the location and aetiology of health and disease that often turns to even more simplistic solutions that are disconnected from wider social, technological, economic, and structural considerations. In doing so, we aim to show that examples, such as Lykon, and the potentially lucrative market of personalised nutritional recommendations is part of a much longer debate over the relationships among food, health, and systems of dietary knowledge and practice.

The article is structured by three sections. First, we give an overview of the emergence of PN since the mid-2000s. Second, we critically examine the claim that PN uniquely tailors dietary guidance to individual physiological needs and requirements by examining the longer history of humoral dietetics. We take this *long durée* approach for two reasons: (i) because PN proponents themselves appeal to this ancient history and we want to critically evaluate their uses of it; and (ii) to show that personalised or individualised approaches to diet pre-date modern dietetics as well as PN. Finally, we critically assess two common assumptions about this history. The first assumes that humoral theory validates contemporary personalised nutrition by providing historical depth and legitimacy; while the second assumes incommensurability between the past and present dietetic regimes. We contend that neither approach is satisfactory and serves to enable questionable practices in the present such as normalising a scientistic understanding of food, deterministic understanding of health that overemphasis individual responsibility for dietary health, and reductively interpreting contemporary dietary practices as disciplinary. We conclude in arguing that a balanced approach to this history is needed; one that neither assumes naïve continuity nor rigid incommensurability.

## On the emergence of modern personalised nutrition

The concept “personalised nutrition” as it is now used emerged in scholarly literature in the mid-2000s. Prior to this there were a handful of references to personalised nutrition as general dietary advice tailored to a specific patient’s needs. For example, in 1988 Crockett et al. proposed the need for “personalized nutrition education” for parents of children considered at-risk of developing cardiovascular conditions (1988). Yet it is this tailoring of general dietary guidance to individual needs, as well as the profession of dieticians, that postgenomic PN either denies or claims that it is wholly distinct from. Since the mid-2000s proponents have emphasised the newness, novelty and promise of PN for specific individuals based on genomic and epigenomic sciences. That is, nutritional information is not merely adapted to an individual’s needs, but nutritional information itself is purportedly derived from an individual’s unique genome, gut-microbiota, and/or lifestyle. As will become apparent below, there is overlap between these two modern iterations of personalised nutrition, however, the latter emphasises its basis in advanced nutritional omic sciences as a point of demarcation.

Notwithstanding the emphasis on advanced sciences, there is no agreed definition for PN. It is often used interchangeably with other terms, such as precision nutrition, nutrigenomics, nutrigenetics, and nutritional genomics. Ordovas et al. observe that definitions tend to focus on outcomes rather than what is actually new or novel about personalised nutrition (2018). For instance, Gibney et al. define it as an approach to assist “individuals in achieving a lasting dietary behaviour change that is beneficial for health” (2016) and Bush et al. state, “experts agree that the goal of PN is to advance human health and wellbeing by tailoring nutrition recommendations and interventions to individuals or groups of individuals with similar traits” (2020). That is, PN is a more individualised or precise approach that will yield better results than previous methods for achieving dietary or health goals. An underlying principle of PN is that individualised nutritional advice will be more effective than general advice. This perspective is captured in the phrase “one size does not fit all”, which has become something of a cliché with multiple articles using some variation of it as a title or to frame their analysis, for examples see (Yeh & Velmahos, 2013; Kramer, 2015; Kolodziejczyk et al., 2019; Fattore et al., 2021; Panagoulias et al., 2021).

Considering attempts to demarcate postgenomic PN from earlier iterations, it is important to examine how and why this newer PN emerged in the mid-2000s. Narratives about the emergence of PN are useful for understanding how proponents frame it in relation to other approaches to nutrition. Reflecting the observation that PN is often defined by its outcomes, a common story of its emergence is that traditional public health interventions using general dietary advice and education have failed to deliver beneficial public health outcomes and change dietary behaviours. Evidence of this purported failure include the obesity epidemic, increase prevalence of type 2 diabetes and other diet-related non-communicable diseases, including mental health conditions. In contrast, the “promise of PN”^1^ is that it is *precise* and tailored to the dietary needs of an individual’s genomic and microbiomic needs. For example, Matusheski et al. summarise the latest developments in PN by noting that “no one diet fits all” and that personalised weight-loss strategies supported by advances in microbiomics “may, in fact, support dietary adherence and long-term weight maintenance” (Matusheski et al., 2021, p. 1493). While acknowledging the need for more research, they conclude that PN can “provide maximum benefit to the individual and advance public health” (Matusheski et al., 2021, p. 1494).

It is worth noting that this framing of PN echoes the centuries old debate between nutrition science and dietetics that often positioned dietetics as practice-oriented feminine care work and lacking in expert knowledge (DeVault, 1995; Brady, 2017). Furthermore, the attempt to demarcate postgenomic PN from traditional public health nutrition rests on two uncharitable interpretations. First, that public health nutrition guidelines had a generalised or “one size fits all” understanding of nutrition. As mentioned in the Introduction, most current nutrition guidelines have different recommendations for different subgroups (Rong et al., 2021). For instance, the Australian Dietary Guidelines have different recommendations based on gender, age, and pregnancy-status (National Health Medical Research Council, 2013; Brownie et al., 2015). In this sense, the modern guidelines provide stratified, not general advice.^2^ This is an interesting aspect with resonances in the longer history of medical humoralism as we shall discuss later. A second uncharitable interpretation used by postgenomic PN is ignoring the skill and expertise of nutritionists and dieticians to apply and adapt the guidelines to the specific needs of their patients and the communities that they work with. Dieticians and nutritionists do not mindlessly apply general recommendations to specific individuals, but work with other health professionals and communities on how best to care for the specific and diverse needs of their patients and clients (Barbour et al., 2022; Boeykens & Van Hecke, 2018; Brady, 2018). Yet proponents of postgenomic PN downplay these nuanced approaches in order to solidify their claim to newness and that “one size does not fit all”.

Importantly, postgenomic PN not only positions itself as the successor to the purported failures of traditional public health nutrition, but as part of a wider response to the underwhelming applications of gene-centric approaches to health associated with the human genome project (HGP). The HGP (1990–2003) operated with the idea that the gene was central to understanding human biology, health and disease. However, as Meloni recounts, “even before the completion of the HGP” molecular biology was beginning to de-center the gene and since 2003 “the gene has come under yet more scrutiny” and its role as an “autonomous agent determining traits and developmental processes becoming more difficult to reconcile with scientific evidence” (Meloni, 2016, p. 190). Not only did it become apparent that the gene did not have the determining effects initially thought, but the “the non-protein coding DNA [was] far from useless” (Meloni, 2016, p. 190). The “junk DNA”, as it came to be known, was recognised as having a regulatory function controlling the activity and expression of genes (Biémont & Vieira, 2006). This re-opened the door for scientists to recognise the regulatory role of the “cellular environment around the DNA, the entire organism, and, in the case of human beings, their social and cultural dynamics” (Meloni, 2016, p. 191). This led to the emergence of related postgenomic (i.e. after the genome) fields, most notably epigenetics, which tries to understand how varying environmental exposures or dietary patterns (i.e. non-genetic factors) can influence gene expression, adaption and development.

While some nutrition scientists continued searching for an “obesity gene” under the gene-centric paradigm (Caro et al., 1996; Rankinen et al., 2006), proponents of postgenomic PN became interested in postgenomic understandings of the interaction among genes, diets, environment, family history and lifestyle. These researchers believe genomic, epigenomic, and microbiomic knowledges provide “relevant information about individuals to deliver more specific healthy eating guidance and other nutritional products and services” (Ordovas et al., 2018). Through these sciences researchers examined different responses to nutrients depending on genotypic and phenotypic characteristics (Frazier-Wood, 2015). However, researchers recognised that it was not only genes or nutrients that needed to be accounted for, but a vast array of exposures shape individual and population health. This led Wild to coin the term exposome. According to Wild, “the exposome encompasses life-course environmental exposures (including lifestyle factors), from the prenatal period onwards” and “is a highly variable and dynamic entity that evolves throughout the lifetime of the individual” (Wild, 2005, p. 1848). Drawing on Wild’s initial formulation Siroux et al note the appeal of the exposome approach, but that it is very complex “both in terms of measuring it (several hundred exposures are to be considered over the life course) and analysing its relationship to health” (Siroux et al., 2016, p. 126). By trying to measure everything, the data sources as well as the scale and scope of interventions dramatically change. As such, it is not just genetic or biological material that matters and requires measurement and analysis, but everything does: exposure to industrial pollutants and domestic cleaning products, early feeding on breast or formula milk, status of microbial colonies, mode of transportation, water quality, diet, tobacco use, climate, and so on.

Yet as Wild noted there is an “imbalance in measurement precision” between the genome and exposome (Wild, 2005, p. 1848). Understanding *everything* as a potential source of exposure has been paralleled by the emergence of the capacity to measure, track and record almost everything via mobile apps and tracking devices. A determining factor for the success or otherwise of PN to provide a more accurate and precise understanding of diet and health is the emergence of “new technology that enables better and continuous measurements of markers of individual health and fitness”, which in turn requires “new analytical tools that interpret this flow of data and transform it into user friendly practical information” (Ordovas et al., 2018, p. 2). As such, smart devices^3^ are increasingly being used by PN researchers as well as artificial intelligence and machine learning (Panagoulias et al., 2021). Not only do these devices enable users to gather large amounts of data in forms that can easily be fed into software programs, but they are purportedly more reliable than self-reporting approaches (Archer et al., 2018). Acknowledging the controversy surrounding such devices, Ordovas et al. believe they could provide sufficient behavioural information to develop algorithms, which also combine biological information, and “may provide a sound basis for personalised recommendations” (Ordovas et al., 2018, p. 2).

Proposed applications and uses of PN can be grouped into three areas. First, to assist individuals with specific diseases or diet-related conditions that would benefit from nutritional support. For example, researchers working on food allergy management believe that personalised nutrition may have a role to play (D’Auria et al., 2019; Ali et al., 2021). Second, to develop effective and precise public health nutrition interventions. As mentioned, an example often used is obesity, but there are also proposals for personalised nutrition to address mental health conditions (Adan et al., 2019). Third, to assist individual’s in achieving goals and desires that may not be directly related to health such as attaining a certain body shape or fulfilling athletic goals. Each of these, particularly the last two, raise epistemological, ethical and political questions that we will return to in the final section.

Many of these applications rely on the synthesis of data to develop behavioral approaches to motivate individuals to change (Macready et al., 2018). Similar to developments in mHealth^4^ and the quantified-self movement, there is an underlying assumption that more data, more information and more measurements naturally lead to more precise, more personalised and more effective interventions (Lupton, 2013; Savard, 2013; Kuch et al., 2020). PN offers new behaviour change techniques that are “data-driven” and “digitised” yet, as will be discussed in the following section, they have earlier precedents in approaches that encouraged individuals to make lifestyle choices and changes (Mayes, 2015). While the techniques might be different and the data more accurate, the proposed interventions ultimately require individuals to choose differently and follow through with those choices. This is what social theorists commonly call responsibilization, or in Ordovas et al’s words, these interventions are “highly dependent on effective collaboration with participants who are being helped to take responsibility for their behaviour, and, ultimately, health” (Ordovas et al., 2018, p. 3). As will be discussed in section three, this has implications for the reconfiguration of determinism in postgenomic sciences and practices.

Like many new areas of science there is excitement for its potential uses. Researchers contend that postgenomic PN can transform public health and address longstanding dietary-related chronic diseases. However, it is not just in public health that there is excitement. Arguably most of the hype is coming from commercial applications, such as Lykon, mentioned in the Introduction. It is not difficult to see the commercial interest in PN as it applies to both diseased and healthy, that is, everyone. But also, it involves an activity that everyone engages in, namely, eating. Unlike other lifestyle and behavioral markers e.g. exercise or smoking, eating is something everyone does. A further major reason for commercial interest in PN is that it presents the possibility for self-enhancement and improvement (Pérez-Troncoso et al., 2021). Numerous companies are offering direct-to-consumer DNA testing to provide dietary and nutrition advice. One company, *myDNA™*, promotes “DNA diet testing for personalised health” plus “nutrition, fitness, and lifestyle optimisation with personalised DNA insights” (myDNA, 2022). Commercial uses of PN are compounded by the ambiguity around the regulation of food and medicine in many countries. That is, the evidence basis of effectiveness and safety of medicines is often higher and more rigorous, while evidence basis for food items purporting to have health benefits is less clear. Food and health companies have taken advantage of this ambiguity for several decades (Gardner, 2006; Mayes, 2015; Marks, 2019; Merrick et al., 2020).

While it is beyond the scope of this paper and our expertise to assess whether or not PN can deliver on its various promises, some researchers within the field have cautioned against the hype (Joost et al., 2007; Stenne et al., 2012). Acknowledging the absence of regulation and “no defined standards”, Grimaldi et al. developed a framework for evaluating science transparent and scientifically sound advice to the public based on nutrigenetic tests (Grimaldi et al., 2017, p. 1). Professional societies have also raised concerns about the potential for preliminary results to be overstated. In 2014, the Academy of Nutrition and Dietetics issued a position statement on nutritional genomics and concluded there was insufficient evidence to “validate that personalized recommendations result in health benefits to individuals and do not cause harm” (Camp & Trujillo, 2014, p. 310). This was followed-up in 2021 by a consensus statement on Incorporating Genetic Testing into Nutrition Care, where again there were questions about evidence and that “it is not yet clear whether incorporating genetic results as an added layer of precision improves nutrition-related outcomes” (Braakhuis et al., 2021, p. 545).

In summary, proponents of PN emphasise the novel ways it can transform individual and population health. They claim that it is a radically new way to understand diet and health, and is developing personalised interventions based on latest postgenomic sciences. However, as discussed above there were immediate precursors to postgenomic PN that tried to tailor nutrition advice to individuals (Crockett et al., 1988). In the following section we further examine the claim of newness by looking at the much longer history dietetics to show that while postgenomic understanding of nutrition is new, the idea and practice of dietary regime tailored to individuals based on seasonal and environmental variants is not.

## Dietetics before modern personalised nutrition

The claim that PN is new appears strongest when compared to late-nineteenth and early-twentieth century attempts to standardise and universalise human diets. Historians consider the mid-nineteenth century as the beginning of modern nutrition science when “laboratory investigations concerning nutritional issues began to gain prominence” in France, Germany and Britain (Kamminga & Cunningham, 1995, p. 3). The development of chemical physiology, and later biochemistry, enabled the identification of key nutritional categories: calories (1819), protein (1838), carbohydrates (1844), and vitamins (1912) (Carpenter, 2003; Hargrove, 2006). This is a complex history entangled with European nationalism, welfarism, agricultural economics and the rise of food corporations among other contingencies (Kamminga & Cunningham, 1995; Harris, 2004; Spary, 2013; Earle, 2019). As noted above, this period marked the beginning of turf wars between nutrition science and dietetics over who had the authority and expertise to provide dietary advice (DeVault, 1995; Cannon, 2005; Shapin, 2014; Brady, 2017, 2018). We cannot enter these complexities here, expect to say that the emergence of modern nutrition science sought to identify a diet comprised of essential nutrients and vitamins for all humans regardless of environmental or cultural contexts (Carpenter, 1988; Scrinis, 2013; Hite, 2018). The imperative to understand the essential dietary requirements for human health led researchers and public health committees to produce dietary guidelines and recommendations they considered applicable to all people (Scrinis, 2013; Mayes & Thompson, 2015; Hite, 2018).

As cited above, PN researchers contend that recommended diets at the population-level have differing effects on individuals and therefore personalised dietary and nutrition recommendations are needed to improve health of the individual. However, as argued, this is not an entirely fair or accurate account. General population guidelines developed in the latter half of the twentieth century were tailored to specific cohorts and subgroups. For example, there has been a history of tailoring nutrition education and interventions to specific groups since 1965 (Crockett et al., 1988; Eyles & Mhurchu, 2009). However, these tailored approached are rarely reflected in the postgenomic PN narrative. Rather, the “one size fits all” paradigm hides and disregards earlier individualised approaches in order to maintain its claim to newness.

A longer view of the history of food, diets, and health, however, shows that twentieth century attempts to standardise dietary guidance, and tailor them to specific subgroups, are themselves part of a complex history of more-than-individual advice within premodern medicine. Certainly, in conceptual terms many have noted the relatively recent (post eighteenth century) emergence of the terms normal, normality and normalization in medical science (Canguilhem, 2007). As the story is often presented, in terms of practices the Hippocratic-Galenic tradition of dietetics resulted from the peculiar understanding of the constant mutability and hence singularity in time and spaces of bodies that made generalizations practically impossible. However, it would be too simplistic to say that ancient and early-modern medical advice didn’t have their own ‘normalizing’ frameworks. Not just because they were embedded in philosophical contexts such as Stoicism first and Christianity (or Islam) later that highlighted moderation and control of passions (Grant, 2002) but also because notions of balance, temperance, and evacuations of bad humours within the same medical framework generated their own normalizing tropes and hence actively shaped political and religious metaphors (Meloni, 2023). Scholarship on dietetic advisory and longevity books that proliferated for European elites with the advent of printing technology and the birth of modern national languages (vernacular) has rightly emphasized the making of an individualistic style (Gentilcore, 2015). However, this understanding must be situated in a wider context of special attention to the dietary requirements of women, different racial groups (including European colonizers in the new colonies), and classes which has always been part of the humoralist tradition (Paster, 1993; Meloni, 2019). Heir to a rich urban family, Galen for instance standardized all bodies and physiologies against “the adult, urban, Greek male, in the prime of life” (Mattern, 2008, p. 105). Peasants for their different diet and lifestyle presented to him a sort of perennial conundrum. They were a class apart, “almost another species” (Mattern, 2013, p. 23, 111), if not true “donkeys in their constitution” (Mattern, 2008, p. 105). Similar comments on the physiological alienness of specific groups such as women or colonized subjects long represented in the fear and anxieties of the humoralist tradition given the alleged perception of a direct effect of food on the humoral and racial constitution (Marwick, 1995; Earle, 2012).

In this section we overview the Hippocratic-Galenic tradition of dietetics to better situate the historical narrative presently used by PN proponents. Examining the way nutrition and dietetic history is used or forgotten in some of its aspects is important for establishing our argument regarding PN and postgenomic determinism. This analysis will also contribute to current scholarship on the importance of history for addressing contemporary problems, from climate science to microbiomics (Wilson, 2013; Dunk et al., 2019; Ludington & Booker, 2019). Before making these arguments in the final section, we trace the development of Hippocratic and Galenic thought and practice, with particular emphasis on the humours and role of the non-naturals.

Hippocratic medicine^5^ or humoralism extends back to the fifth century BC where the notion of a cure through diet (διαιτητική) starts to be explicitly thematized in the so-called Hippocratic corpus (Bartos, 2015). Dietetics is part of a number of emerging soft cures, including cures through waters (hydrotherapia) or herbs/drugs (pharmacopeia), emetics, and venesection (though not common to all schools). These soft cures emerged as alternatives to a more masculine medicine mostly focusing on surgery and cautery for curing military wounds and was for this reason often despised by traditionalists like Plato (Grant, 2002; Meloni, 2019).

Dietetics appears in several passages and is the object of one specific book of the Hippocratic corpus (Περὶ διαίτης, later translated as *De diaeta* in the Latin West). The Hippocratic *Regimen in Health* starts with a very programmatic account of the importance of diet/regimen not just for specific categories of persons (as athletes or soldiers) but for everyone in general:

The layman ought to order his regimen in the following way. In winter eat as much as possible and drink as little as possible; drink should be wine as undiluted as possible, and food should be bread, with all meats roasted; during this season take as few vegetables as possible, for so will the body be most dry and hot. When spring comes, increase drink and make it very diluted, taking a little at a time; use softer foods and less in quantity; substitute for bread barley-cake on the same principle diminish meats, taking them all boiled instead of roasted, and eating when spring comes a few vegetables, in order that a man may be prepared for summer by taking all foods soft, meats boiled, and vegetables raw or boiled taking all foods soft, meats boiled, and vegetables raw or boiled. (1931, p. 45)

The Hippocratic corpus revered the power of the seasons (and all of elements indeed). In *On Regimen*, this changing nature of seasons (quadripartite as for the ecological nature of the Mediterranean basin were these prescriptions firstly emerged in Humoralism) has to be counteracted with a painstaking attention to proper food and wine for each season and indeed time of the day. The nature (*physein*) of the different constitutions of people is also brought into view as a second key factor to coordinate with the first. For instance, the consumption of cucumbers – having a cold and moist quality – would have a different effect on someone with a dominant melancholic (cold and dry) temperament than someone with a phlegmatic (cold and moist) temperament. This careful specificity in the interaction of food properties with humoral qualities produced those meticulously detailed specifications that can be found in later medieval and Renaissance printed dietary or longevity manuals. As Ken Albala notes,

a healthy choleric man (h2, d2) may eat pheasant (h1, d1). The equation would leave him somewhere around hot and dry in the upper first degree, to be precise, the first degree and forty-five minutes. However, garlic (h4, d4) would make him sick, dragging him beyond his natural choleric complexion. But were he distempered (h3, d 3), a cold salad (c 1, m1) would be the perfect corrective. (Albala, 2002, p. 175)

Whereas medieval and later Renaissance handbooks exhibited a peculiar and meticulous specificity, the roots of this “typological” understanding of different humoral temperaments traces back also to the Hippocratic *On Regimen* and later the Galenic On the Powers of Foodstuffs (*De Alimentorum facultatibus*). In the Hippocratic treatise we can read that:

Those with physiques that are fleshy, soft and red, find it beneficial to adopt a rather dry regimen for the greater part of the year. For the nature of these physiques is moist. Those that are lean and sinewy, whether ruddy or dark, should adopt a moister regimen for the greater part of the time, for the bodies of such are constitutionally dry.

Young people also do well to adopt a softer and moister regimen, for this age is dry, and young bodies are firm. Older people should have a drier kind of diet for the greater part of the time […]. (1931, p. 47)^6^

In Galen’s dietary treatise (late second century C.E), similarly, nourishment’s properties (*dynameis*, power, ability, strength) are understood as balancing factors that, cooked via the innate heat of the body (*pepsis*), have to act in the opposite direction of one’s individual temperament (wet, dry, cool or hot), or maintain its average temperament if a body happens to be in that median situation with no prevalence of any specific humour at a certain point of his lifecourse (Grant, 2002). The relationship between bodies and food is also to be understood as two-way: “the effect of particular foods or classes of foods upon the body, and the reciprocal effect of the body upon the foods” (Galen & Powell, 2003, p. 2).

Moving to medieval Europe, the vast archive of books on the rules of health (*regimen sanitatis*) represent a continuous thread connecting the different iterations of humoral medicine across the Mediterranean basin, Islam, and later Latin Europe up to its translation in national languages, and finally the major expansion of the genre after the Renaissance and the first printed health volumes (Albala, 2002). After the tenth century in Islam and twelfth century in Latin West a new genre, *Regimen* or *Tacuinum Sanitatis* become widespread often penned by great medical names such as Arnald de Vilanova or sometimes just anonymous and translated in vulgar: compared to officials manuals of health written specifically for a king or an emperor in the previous centuries, this new medieval companions to health are written in a style that is at the same time direct and capable to be understood and practised by a local population unable to read Latin but wishing to live healthfully (Orofino, 1990; Adamson, 1995; Wallis, 2010).

For instance, an early fourteenth century health manual written in the local Neapolitan language discusses the habits and behaviour of popular classes who cannot access Latin, dispensing advice for the preservation of health, “the common good”, and a “safe living” (*vivere securu*). Food, drinks, and cuisine in general take the lion’s share of the manual with detailed recommendations, to be adjusted with regard to age, season, and temperament, regarding roasting lambs or goats, boiling of fishes, expelling bad humours for meat that needs to be cooked, the mixing of wine and water, the number of grapes to be eaten every morning (28), and the potentially unhealthy nature of other fruits such as melons and white figs (Orofino, 1990, p. 781).

Our reconstruction of this *longue durée* attention to “personalised nutrition” has to be balanced however by a recognition of number of discontinuities with the current emphasis on PN. Firstly, we need to highlight that, in the humoral tradition, whatever the prominence of food and diet, these factors are never understood in isolation from the other factors. In his The Art of Medicine (known as *Ars Medica*) Galen established a very influential distinction between two causes of changes in bodies. Some that are necessary and some that are not. The first included, eating, drinking, breathing, waking and sleeping (and latter emotions or passions of the souls were added). To each of them would correspond “a specific type of healthy cause” (Galen, 1997, p. 375). Those that are not necessary are what we would call today accidents. Galen mentions here “contact with swords and wild beasts” (ibid.). The first “necessary” causes of change is what will then be later codified as in the Middle Ages as the *sex res non-naturales* (six non-naturals). In contrast to *the seven naturals* (physiological aspects such as the four elements, qualities, humours, members and faculties) and the *three contra- naturals* (pathological aspects such as disease, its causes and sequels, which pertained to the doctor’s competence) the *res non naturales* (non- naturals) included all aspects an individual had necessarily to experience and hence could take care of “airs and places; food and drink; exercise; excretion and retention; sleep; and emotions”.

This is to say that, unlike what contemporary PN suggests, a solitary emphasis on only one of the six non naturals, food, without considerations for all the others would make little sense in a humoralist perspective. As historians of the six non-naturals have pointed out when we isolate one of the nonnaturals from the others, abstracting food and drink or any feature of hygienic or therapeutic programmes from all the others, we do some violence to the holistic character of pre-modern medicine (Kennaway & Knoeff, 2020). Physicians from the Ancients to the nineteenth century emphasised that proper regimen took into account all the non-naturals simultaneously (Kennaway & Knoeff, 2020). In this way, the historical emphasis on a holistic approach offers an alternative to over determining the role of food, diets and individual choices. That is, it could provide important lessons to avoid postgenomic forms of determinism present in PN.

A second element of discontinuity pertains to the different ontology of the body between traditional humoralism and our biochemical view of food. To simplify we can say that food mattered more, and in more pervasive way, than for our biochemical model that decompose it in substances. Unlike our modern view of an inner milieu or even an inbuilt genetic system that can relatively buffer the body from environmental stimuli, the direct transformation of food into humours is essential in humoralism. As Ibn Sina notes in his *Canon of Medicine* (which will become part of the syllabus of European medicine until the sixteenth century) a humour “is a nutrient, derived from both food and drink” (paragraph 96). The same metaphor of the body as a great stomach that “concocts” food, maintaining innate heat, and distilling nutrients into various end products, including sexual semen, shed a different light on the power of a properly food to impact directly reproduction and the quality of the offspring. Hence for instance constant advice to fully digest food not just before another meal but also before sexual activity, to avoid monstrous births (Laqueur, 1992).

This connection between food and reproduction becomes particularly vivid and tense since the rise of European colonialism and helps explain European caution and anxiety about embracing new foods and diets of Indigenous populations. While there were overt racialised rejections of Indigenous foodways (Davey et al., 1945; Daigle, 2017; Mayes, 2018), the work of Rebecca Earle highlights the way humoral theory shaped sixteenth century Spanish colonialists attitudes to people, places, and foods in the so-called New World. Food, as discussed above, was one of the key points through which the body and health of the individual could be transformed for good or ill. As such, when the Spanish entered “new environments—whether to a different city or a different continent— which subjected the body to unfamiliar climates and constellations and to unusual foods, therefore required particularly careful attention” (Earle, 2010, p. 695). The idea that bodies – both Indian and European were “mutable and porous, open to the influences of many external forces, including, critically, food” meant that food and diet took a “central place in the maintenance of colonial society” (Earle, 2010, p. 713). Thus in addition to maintaining an individuals health or correcting a humoral imbalance, food was considered a central part of colonial projects in defining and maintaining racialised bodies and identities.

Structural differences, however, between the epistemology of the humoral body and that of the modern body of biomedicine are not enough to minimize the importance that this longer history for the interpretation of the present, particularly in world areas like the Euarasian landmass and South America long shaped by cultures of food as a form of medicine. A first reason for highlighting possible continuities is the epistemic malleability of humoralist medicine and advice, which survived and actually thrived in both the Pagan and the Christian antiquity (Temkin, 1991), was successfully incorporated in Islamic natural philosophy, and then seemed to perfectly suit the aspiration of individualism and control over life variable that started to emerge with early modernity and the Renaissance. A further proof of this chameleon-like capacity of the humoral tradition is its recognized persistence even when mechanistic-Newtonian physics reframed food in terms of original minuscule particles. As historians of the six non naturals note, the formulation of new medical ideas based on mechanics and chemistry during the late seventeenth and eighteenth centuries did not lead to a diminishing importance of the non-naturals, as one would perhaps have been inclined to expect (Galen & Powell, 2003). On the contrary, the Hippocratic emphasis on investigating the endless variety of nature was combined with a renewed stress on observation as the physician’ s only guide made the non-naturals more important than ever (Galen & Powell, 2003; Kennaway & Knoeff, 2020). A second point is the one we have made previously about the slippery terrain of the status of individual advice within humoralist medicine. While it is fair to say that the regulatory ideal of becoming your own physician (by following the six non naturals etc) is part of the pre-eighteenth century medical tradition (Foucault, 2012, p. 35) and possibly contributed to early modern technologies of self-fashioning (see also Coleman, 1974; Schoenfeldt, 1997, 1999), in practice this individualizing ethos clashed against the stratified nature of premodern society in which racial, gendered, and class binaries shaped a quite complex biopolitics when it came to different subgroups (Meloni, 2019).

We believe this complex echo, and conundrum between individuality and group-targeted strategies, albeit in the new language of 21st century molecular medicine, is still very much resonating within current PN attempts. It is important to clarify however that, given the increasingly globalized nature of PN, showing that the idea that nutritional guidance is embedded in a long and complex history of personalisation could have been explored via other sources and in other contexts, such as Ayurvedic tradition. Other potential sources, within a more limited timeframe, could have been the emergence of nutrition science in the nineteenth century and its attempt to displace dietetics and non-allopathic traditions of medicine. These explorations however cannot be dealt within the limited space of this article. In focusing on the history of humoralism, and acknowledging both the strength and limitation of this interpretative approach, we do not wish to detract from other cultural or national contexts. The transformation of the idea of individual-focused nutrition guidance could be fruitfully explored across different social, cultural, geographic and national settings. For instance, the current popularity of PN, wellness culture, and fad diets could be traced through the marginalisation of alternative accounts (e.g. homeopathy and naturopathy) in the nineteenth century via the emergence of biochemistry as the dominant understanding of nutritional heath (Martyr, 2002; Overend, 2020). Likewise, the intersection of religion, science, and wellness, particularly in Anglo-American Protestant sects of the Seventh-Day Adventists and Quakers, provide an additional lineage to explore the historical ebb and flow of the idea of personalised nutrition (Stokes, 2005; Bauch, 2017). However, it is also probably premature to claim that “the long eighteenth century (taking 1840 as terminus ad quem) was in many ways the last age of the six non-naturals” (Kennaway & Knoeff, 2020).

A return in emphasis on the ethics of personalized balance and self-improvement of humoralist medicine seems nowadays part of a threefold awareness that, (a) the ontological or localised view of medicine is unable to deal with major non-communicable disease epidemics (cardiovascular disease, obesity, diabetes etc. Kennaway & Knoeff, 2020); (b) the contemporary neoliberal emphasis on personal self-responsibility which is shaping global health has no difficulty in incorporating (or somehow resonate with) the personal activism and ethics of individual self-fashioning that belong to the humoral tradition of care for the self (Coleman, 1974; Paster, 1993; Schoenfeldt, 1997; Meloni, 2019); finally, (c) a wider return of interest to Hippocratic tropes has emerged in epidemiology in the context of increasing influences of global warming and ecologically driven diseases (Rosenberg, 2012). To this we can add a fourth motive, which is the increasing dismissal of a universalized model of the medical body, insensitive to differences in time, space and ecologies, and the discovery of concepts such as local biologies to describe an ongoing dialectic between bodies and their local context in which both are contingent (Lock, 1993).

Postgenomic disciplines such as epigenetics and microbiomics have had a very specific effect of undermining the timeless mechanistic narratives of bodies (or genomes, and microbiomes) disembedded from time space, personal relationships etc. However, this is not to suggest a neat identity between ancient dietetics and postgenomic personalised nutrition. The relationship of food and medicine is complex and escapes simple answers of linear growth or loss cultural processes; whether they be the growing process of medicalization and rise of biopolitics after the Enlightenment (Foucault), socio-genesis of civilizing manners (Elias) or the myth of a pristine “natural relationship to food” that would be now lost under the restrictions of a uniquely normalizing modernity, to paraphrase Duerr (1998). What we wish to understand in the next section is exactly the possibility of epistemic hybrids and multiple temporalities by which contemporary versions of traditional dietetics hybridize the molecular language of genomics and postgenomics (for instance in epigenetics and microbiomics).

## Politics and uses of nutritional history

In a note in his book on diets and medicine in early modern Europe, Gentilcore speculates that through the emergence of nutrigenomics “we may yet return to this ancient notion” of individualised dietary advice (2015, p. 187 n19). To this point we have tried to show that the claim “one size does not fit all” is not as novel as PN proponents suggest, and that this long history of dietary guidance that in Gentilcore’s words was “intensely individualistic” (2015, p.14) was in fact also constantly on the verge to label and reinscribe through humoral language different groups, races, sexes on the bases of their specific permeability, porosity and vulnerability to the surrounding environment (Paster, 1993). While there are certainly echoes, overlaps and similarities, we are hesitant to suggest that what we are witnessing today can count as a return *tout court* to ancient practices.

Two extremes should be avoided when referring to this complex and context dependent story of food and health in our “age of precision medicine”. On one side, there is an easy validation of contemporary findings in personalised nutrition by placing them under the reassuring authority of Hippocratic writings. This validation often occurs through the misattribution of the quote “let thy food be thy medicine” to Hippocrates (Cardenas, 2013; Morales, 2020). On the other side, we find a clear demarcation that erases the immense archive of conducts, values, and forms of life that represent the often-tacit framework upon which the imperative of healthy food emerges and re-emerges. This approach is used by personalised nutrition researchers, as well as medical sociologists, both of whom wish to emphasis something wholly new in nutrition and the governance of bodies. We discuss the easy validation and the clear demarcation extremes below.

### Easy validation

To sum up, there is a meaningful sense in which we can learn from past attempts to deal with food and the body. However, anachronistic appeals to Hippocratic and humoral dietetics are a shortcut that too easily turns the past into something malleable for the uses of the present. Pete Evans, a celebrity chef, appeals to Hippocrates in promoting his cookbooks, water filtration systems, wellness-sanctuary and other dietary products. His website features, “As Hippocrates, the father of modern medicine, said 2500 years ago, “Let food be your medicine.” I believe food should be our medicine and it should be our first port of call for a healthier life” (Evans, 2022). It is not only celebrity chefs and diet bloggers who appeal to Hippocrates for legitimacy and authority. Cardenas found that between 1983 and 2013 “at least one biomedical journal per year has cited the phrase”(Cardenas, 2013, e260). Today, a Google Scholar search for medical-related articles using the quote post-2013 yields a further 258 results. For example, in a chapter on the role of nutrigenomics in cancer treatment Nepomuceno says,

The importance of nutrition in health is not a new idea. More than two thousand years ago, Hippocrates, the father of Western medicine, wrote: “Let food be thy medicine and medicine be thy food.” What has changed since the time of Hippocrates is our understanding of the details of how nutrition affects our health. (Nepomuceno, 2013, p. 391)

For Nepomuceno the difference between the past and present is a matter of details, rather than different epistemologies and cosmologies. The eradication of difference allows Nepomuceno to outline a linear transition from Hippocrates to the human genome project and then to the emergence of nutrigenomics and how nutrients can alter gene expression. Similarly, in a review article of latest development in the field for the BMJ’s *Frontline Gastroenterology*, Harvey and Neild start with, “the importance of nutrition in health and disease has been recognised from the first publications of medical texts: “Let food be your medicine and medicine be your food”—translated from the Aphorisms of Hippocrates” (2010, p. 19). Like Nepomuceno, they go on to talk about how science as rapidly developed since Hippocrates. The implicit point is that a clear line of medical thought from Hippocrates to the present emphasises the importance of nutrition. More specifically in the example of Harvey and Neild, who were arguing the case for nutrition training in gastroenterology, Hippocrates serves to legitimate their case as the so-called father of medicine noted the importance of nutrition so surely those designing medical curricula should too. These are just two of many similar examples.

The problem with the widespread appeal to “let thy food be thy medicine” is less that it is wrongly attributed to Hippocrates, but as Cardenas demonstrates, “it leads to an essential misconception: in the Hippocratic medicine, even if food was closely linked to health and disease, the concept of food was not confused with that of medicine” (Cardenas, 2013, e260). Food, as discussed in the previous section, was essential to Hippocratic and humoral medicine as it was one of the keyways to respond to imbalances in the body caused by seasons, places, and sex differences. Thus, the power of food was through its enmeshment with an expansive understanding of the permeable interaction among bodies and surrounding world. Furthermore, Hippocratic doctors clearly distinguished food from medicines. While foods with particular humoral properties would assimilate and be “converted into the substance of the body” (Cardenas, 2013, e260), medicines, as understood at the time, would not become part of the body but “*change* the body’s own nature (in terms of humor quality or quantity)” (Cardenas, 2013, e260). Hence the quote misconstrues nature of Hippocratic and humoral medicine.

Correcting the misuse of this quote may seem pedantic, but these easy validation narratives occur at the price of losing the complexity and polysemic meaning of what the entanglement of food-bodies-nature-cosmic forces represented in these different past iterations. We can for instance project on old doctrines our demand for personalised nutrition. However, even when we find recommendations about the specific virtue of some specific foods for some specific category of people (hot or cold, moist or dry, male or female, old or young, people living in certain places rather than others, rarely a single individual) it may always be the case that the medical explanation is ultimately dependent on a wider symbolic system (for instance astrological incompatibility between food and birthdate or the doctrine of signature in medieval medicine) for which the colour of the food or its sociocultural meaning (rabbit for fear, turtledoves for wisdom) is inextricably associated with rational or “medical” explanation. Thus, to imply that there is a linear line from the time of Hippocrates to the present fails to appreciate the complexity and originality with which food and dietetics were understood in the ancient world. Those who take this approach, miss salient lessons for understanding food, diets, and health today, which we will discuss in the final section.

### Demarcation

The clear demarcation approach is also misleading as it eliminates the vast record of behaviours, principles, and ways of living that serve as the frequently unspoken foundation upon which our demand for healthful and nutritious food arises and re-emerges. There are two broad camps that push the incommensurability interpretation: (i) PN proponents emphasising the radical newness of PN due to its unprecedented scientific precision; and (ii) medical sociologists working with a Foucauldian inflected social constructionism. We will start with the latter.

The claim of an incommensurability between the modern experience of food, metabolism, diet, lifestyle, and those of other historical periods has become very popular in certain quarters of the academia after the rise of social constructionism. Under the label of genealogy, a history that proceeds by breaks, ruptures, and conditions of possibility has become increasingly practised in areas like sociology of the body and medicine. In principle, this view began as a salutary reaction to notions of linear growth of knowledge. However, it has often ended in an epistemic insularism (Meloni, 2023). This is the belief what happened to us, the moderns, is unprecedented, new, not translatable, completely different, than other epochs or periods, before the modern state, or modern chemistry, industrialised food, biopolitics and so on. One example of this attitude, pertinent to our story about personalised nutrition, can be found in a “genealogical” article by British medical sociologist David Armstrong’s (Armstrong, 2009) who claims that the idea of health/medically based changeable behaviour is a relatively recent construction whose antecedent can be found in the British journal the Lancet in the 19th century. To quote him;

The “problem” of health-related behaviours, as this paper attempts to show, is relatively recent, in fact barely 40 years old. Its emergence, however, was the result of a series of earlier shifts in perception, language and practice that can be traced to the 19th century. (Armstrong, 2009, p. 910)

This novel language would then stabilize via behaviourism and other recent scientific shift so to underpin the new “core dimension of health and illness” emerging in the 21st century. Of course, none can deny that neither the Hippocratic corpus nor Galen or Ibn Sina, writing in ancient Greek and Arabic respectively, have ever used the word “behaviour” in English or in the sense of the Lancet ca. 1850 that Armstrong sees as unprecedented for the rise of medical surveillance after the nineteenth century. But genealogies that are so narrow in their sources, and reflect so idiosyncratically the location and hence linguistic accessibility for any author, are at best truncated if not ethnocentric. They implicitly promote a disregarding attitude toward the past seen as “no man’s land” or a blank slate where we the moderns can write as we please, what anthropologist Jack Goody has aptly called “the theft of history” (Goody, 2012).

The second response to this history comes from ahistorical and scientistic PN proponents emphasising its newness. That is, the long history of dietetics isn’t actively denied by this group but is not considered remotely relevant. These proponents and researchers, cited in the first section, consider the HGP and twentieth century attempts to standardise diet as the only relevant history to compare and contextualise PN. As outlined in the first section, proposed applications and uses of PN can be grouped into three areas: (i) assist individuals with specific diseases or diet-related conditions that would benefit from nutritional support (e.g. allergy management); (ii) to develop effective and precise public health nutrition interventions (e.g. obesity prevention); and (iii) assist individual’s in achieving goals and desires that may not be directly related to health (e.g. optimizing performance or body shape). Each of these, particularly the last two, raise epistemological, ethical and political questions. A fuller appreciation of the history of bodies, dietetics and health can assist in addressing these questions.

In regard to epistemological questions, the application of PN to address individuals with specifically defined food allergies is different to applications in non-communicable diet-related diseases and conditions, such as obesity. The former is a discrete and known entity with particular causes and remedies, while the later (e.g. obesity) is a complex multi-causal condition that may or may not be directly related to diet. That is, what is known and can be known about these two different areas of PN application need careful qualification, which is often lacking. A clearer awareness of dietetic and nutritional history, not only humoralism, can provide a certain epistemic humility about what is possible.

Furthermore, in ignoring this longer history proponents of PN are able to blithely claim that they have a new approach to diet and health, which serves to give hype and commericalisation a scientistic veneer. Obviously, there are new developments, but claims to scientific advancement is also a strategic move that serves to maintain the idea that PN is evidenced based despite concerns from within the field. As noted above, peak societies like Academy of Nutrition and Dietetics have raised questions here. Evidence base is particularly relevant for the kinds of claims that can be made about individuals. Ordovas et al. note that “most of our evidence in populations is probabilistic. The personalised nutrition approach wants to use this evidence for individuals” (Ordovas et al., 2018, p. 3). This is similar to traditional public health nutrition that seeks to use probabilistic population data about salt intake and heart disease, for example, to then tell individuals that *they* need to reduce their salt intake (Mayes & Thompson, 2015). Ordovas et al. also reach this conclusion in conceding that the “available evidence allows us to predict mean outcomes from a given intervention and genotype, but it is impossible to predict health outcomes for individual” (Ordovas et al., 2018, p. 3). Yet to proceed to tell individuals what to eat based on probabilistic population data is not what PN promised and it is an epistemological leap with ethical consequences by asking individuals to act on advice that is not necessarily relevant or appropriate for them (Mayes, 2015, 2018). As such, current evidence base does not yet support a lot of the PN claims, which is why “other investigators advocate a universal, rather than targeted, approach to lifestyle intervention for disease prevention and treatment” (Ordovas et al., 2018, p. 4).

In addition to epistemological concerns there is a long history of moralised and racialised nutritional advice and its entanglement with politics and economics. As Meloni and others observe, “the moral economy of postgenomics is also neoliberal: it focuses on individual risk” (Meloni, 2016, p. 192). Individual risk and exposures in this neoliberal moral economy often fall along racialised and classed lines (Scrinis, 2008; Guthman, 2011; Goldberg, 2012; Meloni, 2016; Mayes, 2015). This is not only true for PN and postgenomics, but healthcare services and discourses in general (Marmot, 2005; Bond et al., 2020; Russell, 2021; Govindasamy & Carlin, 2022). As Alison Harvey argues, nutrigenomics or PN is not radically new but part of continuum of scientific strategies (2009). For PN to forget or ignore its place in the longer history of moralised and racialised dietary and health guidance risks repeating and perpetuating this past. Its forgetting and overstating the novelty of the present is hence not innocent, but part of the condition of accumulation of a new academic capital based on the cultivated erasure of an ancient ethics of eating with its traditional practices and forms of knowledge, another case of what Proctor and Schiebinger have called agnotology, that is the story of how ignorance is strategically made and unmade as a result of different political struggle and conditions.

### Looking to history for more than demarcation or validation

The power of food and diet to improve or degenerate bodies has diffracted through different nutritional regimes in different societies. Racialised ideals of Europeans “improving” indigenous populations by adopting a “civilised” (i.e. European) diet reverberated in different settler-colonial contexts (Gaskill et al., 2008; Mosby, 2013; Coté, 2016; Whyte, 2017; Mayes, 2018). Today, postgenomic scientific approaches to diet and nutrition, such as PN and gut-microbiomics, provokes similar yet different questions about racial bodies, diets and questions of justice and equality (Benezra, 2020). For instance, in relation to gut-microbes and their influence on individual health, researchers have noted the uneven distribution microbes along race and class lines (Benezra, 2020; Greenhough et al., 2020). If, as PN maintains, that these microbes contribute to health and wellbeing of individuals, then it is necessary to consider the microbial determinants of health and historical uneven distribution of microbes as well as other nutritionally relevant goods. This contributes to an expanded sense of social justice that does not only account for the uneven distribution of education, employment, nutritious food but also the uneven distribution microbes that purportedly contribute to health and wellbeing. This is beyond the scope of the current paper, but we contend that the multiplicity of microbes in and between human agents offers a novel way for re-thinking social justice and countering the dominance of the autonomous individual agent in PN as well as socio-ethical thinking.

Returning to postgenomic determinism, the theme of this special issue, we suggest that appreciating this longer history of individual-focused nutrition guidance provides postgenomic PN with a mirror to reflect on current practices and how an emphasis on individual dietary choices, in the absence of accounting for wider social, economic, microbial and epigenomic can repeat practices that blame, responsiblise, and stigmatise the individual. Through a nuanced appreciation of this history, postgenomic PN has an opportunity to develop a more holistic understanding of health and disease that is more holistic and avoids over-determining individual choices and lifestyles.

The easy validation and incommensurability approaches to this history have implications beyond nutrition popularisers and the tendentious genealogies of medical sociologists. Of course, nutrition researchers did not enter their respective fields to become historians of science. However, the use or denial of this history is not a neutral decision nor just the result of academic silos and “strategic ignorance” among disciplines (McGoey, 2012). It is helpful to sustain the hype of the novelty of the proposed field and its potential commodification of molecular advice that undermines longer histories of food management in premodern and traditional culture. It epitomizes the colonial denial and erasure of what was there before a new civilization, modern science, took place on the immensely rich territory of food practices, microbiopolitics, and forms of knowledge. Moreover, to its own risk and peril, it elides how the longer history of nutritional advice happened in a heavily moralized, classed, gendered and racialized context deeply entwined with politics and economics (Earle, 2010, 2019). Finally, denying this history perpetuates simplistic “scientistic” view of progress and exacerbates contemporary hostilities towards alternative traditions of health and nutrition. It creates cultural binaries whereas *longue durée* histories would relax tension between “Western” and “non Western”, modern and premodern, looking at commonalities, temporal hybrids, and making and remaking of food tropes over mutated sociotechnological circumstances.

## Conclusion

If, as Earle and others suggest, “that the science of food cannot be separated from broader social and political contexts” (Earle, 2019, pp. 81–82), then what do current debates about PN tell us about our current social and political moment? One thing is that the appeal of novelty and precision of postgenomic PN is exciting investors. As noted in the Introduction, Lykon and companies like them, are bringing together nutrigenomics, AI, and hip aesthetics to produce what is ostensibly a weight-loss program aimed at millennials and Gen Z. Yet Lykon is careful to avoid such associations. Their marketing materials and website emphasises that dietary recommendations are personalised and based in the genomic sciences. This is captured by their slogan “Honest Science. Made for You” (Lykon, 2022).

Despite significant commercial interest, researchers in the field of public health nutrition and PN are often driven by a desire to improve the health of individuals and populations. Ordovas et al., for instance, explicitly state their concern about how to best focus on individuals to improve population health without increasing health disparities (Ordovas et al., 2018). Yet, the influence of commercial forces in the application of PN, as well as the general methodological individualism that public health interventions are shaped by (Goldberg, 2012), make the quest for health equity seem improbable. However, there are cracks of possibility. Earle contends that the development of nutrition science from the end of the nineteenth century onwards led to the “progressive excision of embodied knowledge” of actual people who eat to be replaced by a disembodied “set of scientific facts” (Earle, 2019, p. 83). There is clearly a continuation of this trend within PN that focuses in on the molecular and microbial level that neglects the historical, environmental, social and racial contexts in which the bodies hosting those molecules and microbes exist (Landecker, 2011). However, there is also the possibility for re-embodying nutritional knowledges that re-admits the body, environments, and history into our understanding of food, diets and health. This has resonance with older public health ideas of the social determinants of health and the pursuit of health justice. But this requires PN and related areas such as human-gut microbiomics to open the door for new kinds of embodied and individualised knowledges (Lappé, Hein, & Landecker, 2019; Benezra, 2020; Greenhough et al., 2020; Ishaq et al., 2021; Raffaetà, 2022).

In response the individualising effects of personalised medicine, of which PN is a subset, Prainsack argues for the need for solidarity (Prainsack, 2018). Landecker has analysed the way this expansive conception of exposure also significantly changes our understanding and relationship to food (Landecker, 2011). That is, PN and associated areas of nutritional epigenetics and gut-microbiomics, position food again as a locus for reconceiving socially and embodied connectedness, notions of responsibility and the possibility of fostering equitable food and health environments. Yet, we contend that it is also important to remain wary about these possibilities and associated theorisations. As Landecker notes in relation to the ‘hopeful narratives’ of nutritional epigenetics, the sites and logics of hope also hold the “potential reinscriptions of social, economic and cultural difference” (Landecker, 2011, p. 179). The point of drawing attention to this history is not merely to deflate the hype surrounding modern PN, or acknowledge subaltern traditions of knowledge, but to put PN in its appropriate historical context. As food, history too is a good medicine, as it helps avoid the convenient making of simplistic causal accounts, linear historical affiliations, and a blindness to the unavoidable connection between technologies of the self and biopolitics of groups which we have found operating across the longer history traced in this article.

### Electronic supplementary material

Below is the link to the electronic supplementary material.

Supplementary Material 1

## Footnotes

1 This is another cliched phrase used in numerous articles. Examples include (Ferrario et al., 2021; Ordovas & Berciano, 2020; O’Donovan et al., 2016).

2 We thank the anonymous reviewer for pushing us to consider this point.

3 Devices like phones that connect with the internet and other networks to share and receive data and information.

4 mHealth is an abbreviation of “mobile health”. It is the use of smart devices, such as phones and tablets, for the purpose of improving, monitoring, or reporting on health behaviours and status.

5 By Hippocratic tradition we do not mean in a naively presentist way the origins of “Western” medicine, but a wider network of ontologies and practices of the body (individual and collective) that characterized whole civilizations in the AfroEurasian landmass for centuries, from Egypt to India to North Africa and the Iberian Peninsula, even before Hippocratic writings were systematized in the fifth and fourth century BCE.

6 A typological partition of people according to humours is also evoked in Ayurveda to distinguish types of food for different metabolic temperaments *Vata*are irregular metabolizers, *Pitta* are fast metabolizers, and *Kapha* are slow metabolizers (Rastogi, 2014). We lack space in this article to address in any serious way analogies and differences of dietetics in Hippocratic and Islamic, Indian, Chinese and South East Asians systems of medicine. See for instance, (Gwei-Djen & Needham, 1951; Kastner, 2004; Waines, 1999; Banamali, 2014; Banerjee, Debnath & Debnatth, 2015; Scheid, 2020).
