# Supplementary material for: Forgetting how we ate: personalised nutrition and the strategic uses of history
Source: Hist Philos Life Sci. 2024 Mar 7;46(1):14. doi: 10.1007/s40656-024-00613-x (PMC10920492; doi:10.1007/s40656-024-00613-x)
Supplement: Supplementary file 1 — Supplementary Material 1 [file 40656_2024_613_MOESM1_ESM.docx]

| Editor comments | Response |
| --- | --- |
| « microbiomic profile »: better change to microbial profile | Changed |
| “We take this long durée approach for two reasons: i) because PN proponents themselves appeal to this ancient history and we want to critically evaluate their uses of it; and ii) to show that personalised or individualised approaches to diet pre-date modern dietetics as well as PN. Finally, we critically assess two common responses to this history: i) to assume that humoral theory validates contemporary personalised nutrition by providing historical depth and legitimacy; and ii) to assume incommensurability between the past and present dietetic regimes.”  - Use two different kinds of numeration, i, ii, 1 and 2 or similar.  - This could also be mixed up by section numbers i, ii, iii. Fit these section numbers to HPLS style, i.e. 1, 2, 3… | We have followed the HPLS guidelines for section numbers. E.g. 1, 1.1 etc  And in the section identified here we have converted the second set of “i) and ii)” to text (see highlighted section). |
| “As noted above, it also a period the marked the beginning of turf wars between nutrition science and dietetics over who had the authority and expertise to provide dietary advice (Brady 2017, 2018; Cannon 2005; DeVault 1995; Shapin 2014).”  Check sentence. Also after this sentence (the dot), there is a tab stop missing. | Sentence modified to: “As noted above, this period marked the beginning of turf wars between nutrition science and dietetics over who had the authority and expertise to provide dietary advice (Brady 2017, 2018; Cannon 2005; DeVault 1995; Shapin 2014).”  Tab stop included. |
| Check all in text references for correct HPLS format. There are several different formats used. Check esp. for “,” “and/&”, “;” and “p.”. | Checked and corrected all in-text references. |
| Check all in text references: some like “Davey, Macpherson, and F.W. 1945” are incomplete or need to be changed. | Checked and corrected in accordance with HPLS style guide |
| “…Hippocratic corpus (Bartos 2015).“  add tab stop. | Added |
| Check format of block quotes:  “…Older people should have a drier kind of diet for the greater part of the time (….) (Regimen in Health, 4, Loeb, p. 47)6.”  Change to “…Older people should have a drier kind of diet for the greater part of the time […]. (Regimen in Health, 4, Loeb, p. 47)6”  - Also Hippocrates/Loeb is missing from the bibliography.  - Also check the place of dots in all block quotes (at some point, e.g., p. 20, you place the dot before the brackets). It should be quote, dot, bracket, no dot. | Corrected throughout. |
| longue durée is sometimes italicized, sometimes not. Make consistent. | Corrected to be italicized throughout. |
| “…in the new language of 21st molecular medicine…” --> 21st century | corrected |
| “…Raffaeta' 2022).“ Check name. | corrected |
| Check bibliography to match HPLS style and consistency (e.g. abbreviations of first names, lower case letters, etc.). | Checked and corrected in accordance with HPLS style guide |
